# Bioactive oat β-glucan reduces LDL cholesterol in Caucasians and non-Caucasians

**DOI:** 10.1186/1475-2891-10-130

**Published:** 2011-11-25

**Authors:** Thomas MS Wolever, Alison L Gibbs, Jennie Brand-Miller, Alison M Duncan, Valerie Hart, Benoît Lamarche, Susan M Tosh, Ruedi Duss

**Affiliations:** 1 Glycemic Index Laboratories, Inc., Toronto, Ontario, Canada; 2 Department of Statistics, University of Toronto, Toronto, Ontario, Canada; 3 School of Molecular Bioscience, University of Sydney, NSW, Australia; 4 Human Nutraceutical Research Unit, University of Guelph, Guelph, Ontario, Canada; 5 Reading Scientific Services, Ltd, Reading, Berkshire, UK; 6 Institute on Nutraceuticals and Functional Foods, Université Laval, Québec, Canada; 7 Agriculture and Agri-Foods Canada, Guelph Food Research Centre, Guelph, Ontario, Canada; 8 CreaNutrition AG, Zug, Switzerland

**Keywords:** Randomized clinical trial, oats, beta-glucan, ethnicity, cholesterol

## Abstract

**Background:**

There is increasing global acceptance that viscous soluble fibers lower serum LDL cholesterol (LDL-C), but most evidence for this comes from studies in Caucasians. To see if oat β-glucan lowers LDL-C in Caucasians and non-Caucasians we conducted a post-hoc analysis of the results of a randomized, controlled, double-blind, multi-center clinical trial whose primary aim was to determine if molecular-weight (MW) influenced the LDL-C-lowering effect of oat β-glucan.

**Results:**

Caucasian and non-Caucasian subjects with LDL-C-C ≥ 3.0 and ≤ 5.0 mmol/L (n = 786 screened, n = 400 ineligible, n = 19 refused, n = 367 randomized, n = 345 completed, n = 1 excluded for missing ethnicity) were randomly assigned to consume cereal containing wheat-fiber (Control, n = 74:13 Caucasian:non-Caucasian) or 3 g high-MW (3H, 2,250,000 g/mol, n = 67:19), 4 g medium-MW (4 M, 850,000 g/mol, n = 50:17), 3 g medium-MW (3M, 530,000 g/mol, n = 54:9) or 4 g low-MW (4 L, 210,000 g/mol, n = 51:12) oat β-glucan daily for 4 weeks. LDL-C after 4 weeks was influenced by baseline LDL-C (p < 0.001) and treatment (p = 0.003), but not ethnicity (p = 0.74). In all subjects, compared to control, 3 H, 4 M and 3 M reduced LDL-C significantly by 4.8 to 6.5%, but 4 L had no effect. Compared to control, the bioactive oat β-glucan treatments (3H, 4M and 3M) reduced LDL-C by a combined mean (95% CI) of 0.18 (0.06, 0.31) mmol/L (4.8%, n = 171, p = 0.004) in Caucasians, a value not significantly different from the 0.37 (0.09, 0.65) mmol/L (10.3%, n = 45, p = 0.008) reduction in non-Caucasians.

**Conclusion:**

We conclude that oat β-glucan reduces LDL-C in both Caucasians and non-Caucasians; there was insufficient power to determine if the magnitude of LDL-C-lowering differed by ethnicity.

**Trial Registration:**

ClinicalTrials.gov: NCT00981981

## Introduction

Coronary heart disease (CHD) is a leading cause of morbidity and mortality globally [1,2]. It is well established that reducing serum low-density-lipoprotein-cholesterol (LDL-C) reduces risk for CHD [3]. Lifestyle modifications to reduce CHD risk include increased consumption of viscous soluble dietary fiber [4]. Oats are a good source of soluble fiber, the major component of which is (1→3)(1→4)-β-D-glucan, or β-glucan. Many, but not all studies have shown that oat-containing food products reduce serum LDL-C [5]. We showed that the LDL-C-lowering effect of oat β-glucan depended on its viscosity [6] which, in turn, depends on molecular weight (MW) and solubility [7]; 3-4 g/d of oat β-glucan with MW ranging from 5.3 × 10^5 ^to 2.2 × 10^6 ^g/mol significantly reduced LDL-C by 4.8 to 6.5%, but 4 g/d of oat β-glucan with MW 2.1 × 10^5 ^g/mol had no significant effect [6]. Since there is evidence that the prevalence of blood lipid abnormalities and other CHD risk factors varies in different ethnic groups [8] and that the LDL-C-lowering effect of statins differs by ethnicity [9,10], we examined our data to see if the effect of oat β-glucan on LDL-C varied by ethnicity.

## Methods

The methodology and main results of the trial have been reported elsewhere [6]. Briefly, we conducted a double-blind, randomized, parallel design, controlled clinical trial at 2 contract-research-organizations and 3 university nutrition research centers. Participants were healthy men and women aged 35 to 70 yr with body mass index (BMI) ≥ 18.5 and ≤ 40.0 kg/m^2 ^and fasting serum LDL-C ≥ 3.0 and ≤5.0 mmol/L; 786 subjects were screened, 400 did not meet the inclusion criteria, 19 declined to participate, 367 were randomly assigned to one of the 5 treatments and 22 discontinued treatment before the end of the trial. Written informed consent was obtained from all subjects. The protocol was approved by the ethics review committee at each participating institution. The trial was registered at http://www.clinicaltrials.gov with identifier number NCT00981981.

After eligibility had been determined subjects, stratified by center and by LDL-C (stratum; low, 3.0 ≤ LDL-C ≤ 3.8 mmol/L; or high, 3.8 < LDL-C ≤ 5.0 mmol/L), were randomly assigned to receive a ready-to-eat wheat bran cereal (Control), or oat cereal providing a total of 3 g High-MW (3 H, MW = 22.5 × 10^5 ^g/mol), 4 g Medium-MW (4 M, MW = 8.5 × 10^5 ^g/mol), 3 g Medium-MW (3 M, MW = 5.3 × 10^5 ^g/mol) or 4 g Low-MW (4 L, MW = 2.1 × 10^5 ^g/mol) oat β-glucan per day for 4 weeks. Cereals were packed into air-tight foil sachets each containing half the daily dose (10-14 g) and labelled with a code. After the baseline blood sample, subjects were given a 1-week supply (14 sachets) of their assigned cereal and instructed to consume 2 sachets daily, one with breakfast and one with another meal or snack. Otherwise, subjects maintained their usual diets and other lifestyle habits throughout the study. The daily dose of cereals contained 234-339 kJ, 3-4 g protein, 1 g fat, 9-13 g carbohydrate and 6-8 g total fiber [6]. Fasting blood was obtained weekly for analysis of total and HDL cholesterol, triglycerides and calculated LDL-C.

Here we report the results for LDL-C in subjects based on their ethnicity. Ethnicity was determined based on the subjects' own classification of themselves as Caucasian, Black, Aboriginal, South Asian, Arab/West Asian, Filipino, South East Asian, Hispanic, Chinese, Japanese, Korean or other. Since the number of subjects in each non-Caucasian category was too small for meaningful comparisons, for the purposes of this analysis, subjects were classified as either Caucasian or non-Caucasian.

Statistical analyses were performed using SAS (SAS 9.2, XP-Pro, 2002-2008, SAS Institute, Inc., Cary, NC) on an intent-to-treat basis using all available data from the 366 subjects randomly assigned to treatments and whose ethnicity could be identified (one of the 367 randomized subjects was excluded for missing data on ethnicity). For all 5 treatments, the main effects of treatment and ethnicity and the treatment × ethnicity interaction on week 4 LDL-C was examined using analysis of covariance (ANCOVA), controlling for baseline LDL-C and stratum; the effects of sex, center, age, waist circumference and BMI were tested but not included in the final model because they had no significant effect (P > 0.1). Post hoc testing was performed after demonstration of significant heterogeneity by ANCOVA using Fisher's LSD. The criterion for declaring a statistically significant difference was 2-tailed p < 0.05.

## Results

**Figure 1:**
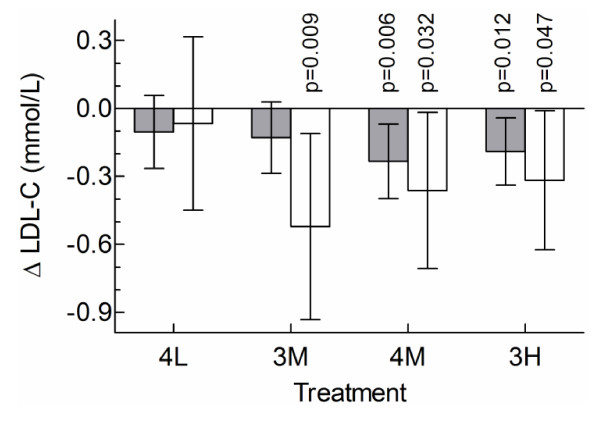
**Time course of unadjusted percent changes from baseline of LDL-C in Caucasian (C) and non-Caucasian (N) subjects randomly assigned to consume the control cereal (Cont, C:N n = 74:13), or cereals containing 4 g low-MW oat β-glucan (4 L, C:N n = 51:12), 3 g medium-MW oat β-glucan (3 M, C:N n = 54:9), 4 g medium-MW oat β-glucan (4 M, C:N n = 50:17) or 3 g high-MW oat β-glucan (3 H, C:N n = 67:19) daily for 4 weeks**. Values represent means ± SEM.

**Figure 2:**
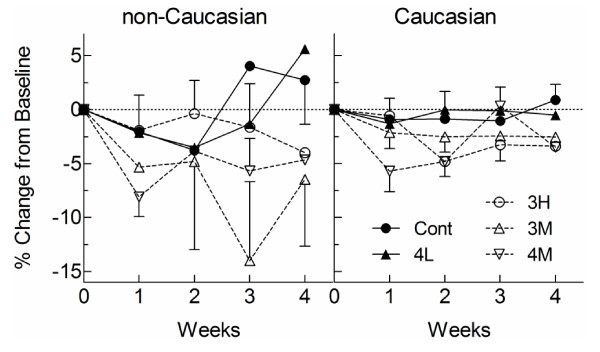
**Difference in LDL-C, adjusted for baseline, in Caucasian (C, filled bars) and non-Caucasian (N, open bars) subjects after 4 weeks on cereals containing 4 g low-MW oat β-glucan (4 L, C:N n = 51:12), 3 g medium-MW oat β-glucan (3 M, C:N n = 54:9), 4 g medium-MW oat β-glucan (4 M, C:N n = 50:17) or 3 g high-MW oat β-glucan (3 H, C:N n = 67:19) daily versus mean adjusted-LDL-C after 4 weeks on Control in the same ethnic group**. Values are means ± 95% CI.

The 296 Caucasian participants were older than the 70 non-Caucasians but were similar in sex, BMI and blood lipids at baseline (Table 1). The total number (% non-Caucasian), respectively, assigned to the 5 treatments were: Control, 87 (15%); 3 H, 86 (22%), 4 M, 67 (25%); 3 M, 63 (14%), and 4 L 63 (19%) (p = 0.39). For most, but not all, treatments, LDL-C tended to respond within 1-2 weeks of starting treatment and remain stable over the last 2 weeks of the study (Figure 1). The results for ANCOVA for LDL-C at week 4 showed significant effects of baseline LDL-C (p < 0.001), stratum (p < 0.001) and treatment (p = 0.003) but no significant effect of ethnicity (p = 0.74) and no treatment × ethnicity interaction (p = 0.34). The latter indicates that the effects of the 5 treatments on LDL-C did not differ significantly in Caucasians vs non-Caucasians. Compared to Control, 3 H and 4 M reduced LDL-C significantly in Caucasians and 3 H, 3 M and 4 M reduced LDL-C significantly in non-Caucasians (Figure 2). 4 L had no effect in either group. When the results of the bioactive treatments (3 H, 4 M and 3 M) were pooled and compared to control, bioactive oat β-glucan reduced LDL-C by a mean (95% CI) of 0.18 (0.06, 0.31) mmol/L (4.8%, p = 0.004) in Caucasians and by 0.37 (0.09, 0.65) mmol/L (10.3%, p = 0.008) in non-Caucasians; the difference between Caucasians and non-Caucasians was not significant (p = 0.24).

**Table 1:** Characteristics of Caucasian and Non-Caucasian subjects at baseline.

	Caucasian		Non-Caucasian		
n (male:female)	131:165		30:40		ns
	Mean	SD	Mean	SD	p
Age (yr)	53.5	9.1	47.0	7.9	< 0.001
Body Mass Index (kg/m^2^)	27.5	4.2	27.5	4.3	ns
Waist circumference (cm)	93.3	21.1	90.8	12.2	ns
Total cholesterol (mmol/L)	5.99	0.72	5.77	0.77	ns
Triglycerides (mmol/L)	1.54	0.84	1.58	0.93	ns
HDL cholesterol	1.49	0.41	1.38	0.40	ns
LDL cholesterol	3.78	0.68	3.64	0.82	ns

## Discussion

There is concern that the burden of chronic diseases is neglected globally especially in low- and middle-income countries [11]. Serum cholesterol, a marker of CHD risk, has fallen over the last 30 years in high-income countries but has not changed significantly or even risen in low- and middle-income countries [12]. To tackle the burden of chronic disease a combination of strategies may be used to promote healthy lifestyles [13]. It was suggested that private-sector initiatives (such as high-fiber breakfast-cereals) may be useful but more evidence on their effectiveness is needed [13]. In this context, there is increasing acceptance that viscous soluble fiber lower serum cholesterol, with claims allowed in Malaysia for products containing oat β-glucan [14] and Japan for products containing psyllium [15]. However, the evidence for these effects comes largely from studies in Caucasians. Thus, our results showing that oat β-glucan lowered LDL-C in non-Caucasians may be helpful to support global efforts to reduce CHD risk.

Since our study was not designed to compare the LDL-C-lowering effect of oat β-glucan in different ethnic groups, the results should be interpreted with caution. Also, we pooled non-Caucasian ethnic groups amongst whom differences may exist. Nevertheless, with 19% of subjects being non-Caucasian, few, if any studies to date on the cholesterol-lowering effects of oat β-glucan include a population as large and diverse as ours. Bioactive oat β-glucan tended to lower LDL-C more in non-Caucasians than Caucasians (0.37 vs 0.18 mmol/L; or 10.3 vs 4.8%), but the difference was not statistically significant. However, the study only had 34% power to detect a significant difference between Caucasians and non-Caucasians and, thus, was underpowered. Nevertheless, our results are consistent with the tendency for 3 g oat β-glucan in a ready-to-eat cereal to reduce LDL-C more in Hispanic Americans [16] than in predominantly (86%) Caucasian subjects [17], 7.5 vs 4.4% relative to control. Therefore, this effect may warrant further study.

## Conclusion

We conclude that oat β-glucan reduces LDL-C in Caucasians and non-Caucasians, but there was insufficient power to determine if the magnitude of LDL-C-lowering differs by ethnicity.

## Competing interests

TW is president of Glycemic Index Laboratories, Inc.; VH is employed by Reading Scientific Services, Ltd.; RD is employed by CreaNutrition, AG.

## Authors' contributions

RD and ST obtained funding for the study; TW, ST and AG conceived of and designed the study; TW, JB-M, AD, VH and BL supervised the acquisition of the data; TW and AG analyzed and interpreted the data; TW drafted the manuscript; all authors contributed to the design of the study, reviewed the manuscript for important intellectual content and read and approved the final version.

## References

[B1] Murray CJL Lopez AD Mortality by cause for eight regions of the world: Global Burden of Disease Study Lancet 1997 349 1269 1276 10.1016/S0140-6736(96)07493-4 9142060

[B2] Murray CJL Lopez AD Regional patterns of disability-free life expectancy and disability-adjusted life expectancy: Global Burden of Disease Study Lancet 1997 349 1347 1352 10.1016/S0140-6736(96)07494-6 9149696

[B3] Delahoy PJ Magliano DJ Webb K Grobler M Liew D The relationship between reduction in low-density lipoprotein cholesterol by statins and reduction in risk of cardiovascular outcomes: an updated meta-analysis Clin Thera 2009 31 236 244 10.1016/j.clinthera.2009.02.017 19302897

[B4] Bazzano LA He J Ogden LG Loria CM Whelton PK Dietary fiber intake and reduced risk of coronary heart disease in US men and women: the National Health and Nutrition Examination Survey I Epidemiologic Follow-up Study Arch Intern Med 2003 163 1897 1904 10.1001/archinte.163.16.1897 12963562

[B5] Brown L Rosner B Willett WW Sacks FM Cholesterol-lowering effects of dietary fiber: a meta-analysis Am J Clin Nutr 1999 69 30 42 9925120 10.1093/ajcn/69.1.30

[B6] Wolever TMS Tosh SM Gibbs AL Brand-Miller J Duncan AM Hart V Lamarche B Thomson BA Duss R Wood PJ Physicochemical properties of oat β-glucan influence its ability to reduce serum LDL cholesterol in humans: a randomized clinical trial Am J Clin Nutr 2010 92 723 732 10.3945/ajcn.2010.29174 20660224

[B7] Wood PJ Beer MU Butler G Evaluation of role of concentration and molecular weight of oat β-glucan in determining effect of viscosity on plasma glucose and insulin following an oral glucose load Brit J Nutr 2000 84 19 23 10961156

[B8] O'Meara JG Kardia SLR Armon JJ Brown CA Boerwinkle E Turner ST Ethnic and sex differences in the prevalence, treatment, and control of dyslipidemia among hypertensive adults in the GENOA study Arch Int Med 2011 164 1313 1318 10.1001/archinte.164.12.1313 15226165

[B9] Simon JA Lin F Hulley SB Blanche PJ Waters D Shiboski S Rotter JI Nickerson DA Yang H Saad M Krauss RM Phenotypic predictors of response to simvastatin therapy among African-Americans and Caucasians: the Cholesterol and Pharmacogenetics (CAP) Study Am J Cardiol 2006 97 843 850 10.1016/j.amjcard.2005.09.134 16516587

[B10] Mangravite LM Medina MW Cui J Pressman S Smith JD Rieder MJ Guo X Nickerson DA Rotter JI Krauss RM Combined influence of LDLR and HMGCR sequence variation on lipid-lowering response to simvastatin Arterioscler Thromb Vasc Biol 2010 30 1485 1492 10.1161/ATVBAHA.110.203273 20413733 PMC2909117

[B11] Geneau R Stuckler D Stachenko S McKee M Ebrahim S Basu S Chockalingham A Mwatsama M Jamal R Alwan A Beaglehole R Raising the priority of preventing chronic diseases: a political process Lancet 2010 376 1689 1698 10.1016/S0140-6736(10)61414-6 21074260

[B12] Farzadfar F Finucane MM Danaei G Pelizzari PM Cowan MJ Paciorek CJ Singh GM Lin JK Stevens GA Riley LM Ezzati M Global Burden of Metabolic Risk Factors of Chronic Diseases Collaborating Group (Cholesterol) National, regional, and global trends in serum total cholesterol since 1980: systematic analysis of health examination surveys and epidemiological studies with 321 country-years and 3.0 million participants Lancet 2011 377 578 586 10.1016/S0140-6736(10)62038-7 21295847

[B13] Cecchini M Sassi F Lauer JA Lee YY Guajardo-Barron V Chisholm D Tackling of unhealthy diets, physical inactivity, and obesity: health effects and cost-effectiveness Lancet 2010 376 1775 1784 10.1016/S0140-6736(10)61514-0 21074255

[B14] Food Safety & Quality Division, Ministry of Health Malaysia Guide to nutrition labelling and claims 2010 http://fsq.moh.gov.my/v2/modules/xt_conteudo/index.php?id=30

[B15] Ohama H Ikeda H Moriyama H Bagchi D Health foods and foods with health claims in Japan Nutraceutical and Functional Food Regulations in the United States and Around the World 2008 Massachusetts: Academic Press 251 280

[B16] Karmally W Montez MG Palmas W Martinez W Branstetter A Ramakrishnan R Holleran SF Haffner SM Ginsberg HN Cholesterol-lowering benefits of oat-containing cereal in Hispanic Americans J Am Dietet Assoc 2005 105 967 970 10.1016/j.jada.2005.03.006 15942550

[B17] Maki KC Beiseigel JM Jonnalagadda SS Gugger CK Reeves MS Farmer MV Kaden VN Rains TM Whole-grain ready-to-eat oat cereal, as part of a dietary program for weight loss, reduces low-density lipoprotein cholesterol in adults with overweight and obesity more than a dietary program including low-fiber control foods J Am Dietet Assoc 2010 110 205 214 10.1016/j.jada.2009.10.037 20102847

